# Supplementary material for: Nondestructive Larval Genotyping of Danio rerio for Mitochondrial and Nuclear DNA Genetics
Source: Zebrafish. 2025 Sep 5;22(6):197–207. doi: 10.1177/15458547251379703 (PMC13671266; doi:10.1177/15458547251379703)
Supplement: Supplementary file 1 [file sj-docx-2-zbf-10.1177_15458547251379703.docx]

**Supplementary Figure 1:** **Effect of 5% non-fat dry milk (NFDM) solution on larval survival post genotyping.** **(A)** Survival percentage of wild type larvae at 24 hours post genotyping in 96-well plate treated with NFDM, 96-well plate without 5% NFDM and unshaken control group. Bars represent survival percentage from three independent genotyping experiments. p-Values were determined by Student's t-test (*p < 0.05; **p < 0.01; ***p<0.001). (N=60 technical replicates across 3 experiments). **(B-D)** Representative bright-field images of 3-dpf wild type larvae: **(B)** unshaken control, **(C)** 96-well plate without 5% NFDM showing tail damage in subset of larvae, and **(D)** 96-well plate with 5% NFDM showing undamaged, intact larvae. (Scale bar: 500 μm). **(E-F)** Gel electrophoresis demonstrating that 5% NFDM pre-treatment to 96-well plates does not interfere with the mitochondrial DNA genotyping, showing successful amplification of mitochondrial genes from larvae in 5% NFDM-treated 96-well plates: **(E)** *mt-cytb* (460 bp) and **(F)** *mt-tl1* (363 bp).
