# Supplementary material for: Nondestructive Larval Genotyping of Danio rerio for Mitochondrial and Nuclear DNA Genetics
Source: Zebrafish. 2025 Sep 5;22(6):197–207. doi: 10.1177/15458547251379703 (PMC13671266; doi:10.1177/15458547251379703)
Supplement: Supplementary file 2 [file sj-docx-3-zbf-10.1177_15458547251379703.docx]

**Supplementary Figure 2:** **Mitochondrial DNA Amplification for zebrafish *mt-atp6* and *mt-co1*. (A)** Gel electrophoresis of the mitochondrial gene *mt-atp6*, showing amplification of the 369 bp band. **(B)** Gel electrophoresis of the mitochondrial gene *mt-co1*, showing amplification of the 494 bp band.
