# Supplementary material for: Nondestructive Larval Genotyping of Danio rerio for Mitochondrial and Nuclear DNA Genetics
Source: Zebrafish. 2025 Sep 5;22(6):197–207. doi: 10.1177/15458547251379703 (PMC13671266; doi:10.1177/15458547251379703)
Supplement: Supplementary file 3 [file sj-docx-4-zbf-10.1177_15458547251379703.docx]

**Supplementary Figure 3:** **Volcano plot illustrating differentially expressed genes.** The x-axis represents the log_2_ fold change in gene expression, with positive values indicating upregulation and negative values indicating downregulation. The y-axis shows the minus log_10_ of the adjusted p-value, representing statistical significance. Each dot represents a gene, with black dots indicating genes not significantly changed and green dots indicating significantly upregulated genes above the threshold (dotted line at adjusted p-value = 0.05).
