# Supplementary material for: Nondestructive Larval Genotyping of Danio rerio for Mitochondrial and Nuclear DNA Genetics
Source: Zebrafish. 2025 Sep 5;22(6):197–207. doi: 10.1177/15458547251379703 (PMC13671266; doi:10.1177/15458547251379703)
Supplement: Supplementary file 4 [file sj-docx-5-zbf-10.1177_15458547251379703.docx]

**Supplementary Materials**

**Supplementary Table 1:** List of oligonucleotides used in this study

| **Target gene** | **Forward primer (5**′ **-3**′**)** | **Reverse primer (5**′**-3**′**)** |
| --- | --- | --- |
| *mt-nd1* | CAGGAAGACACATGACTTCTA | AGTTGGTCGTATCGGAATCG |
| *mt-cytb* | GCAATACACTACACCTCAGACAT | ATCCGGTTTCGTGGAGAAATAG |
| *mt-rnr1* | GGTTAAACGAGAGGCTCTAGT | ATTCCTTCACAGGGTAAGCTG |
| *mt-tl1* | GTTCAGACCGGAGTAATCCA | GGAGTTGTATATAGCCTAACAC |
| *mt-atp6* | ACCGTCCAAACATGATTGACT | TGCTGTGAGGTTTGCAGTAAG |
| *mt-co1* | GGAAACTGACTTGTGCCACT | ACTTCTGGGTGGCCAAAGAA |
| *mavs* | GGCCGTATATGGGTCGTTTT | TTGTGATCCCCCTGATCCTG |
| *cox7b* | CGTAAAACCACGGAACAGTTT | GGAAAGTCCATTTGATAGTTGACC |
| *lrpprc* | CAAGTCCTCACATTTGTTACC | CTTTCACTGCAGACATGTGG |
